# Brief Mindfulness Breathing Exercises and Working Memory Capacity: Findings from Two Experimental Approaches

**DOI:** 10.3390/brainsci11020175

**Published:** 2021-02-01

**Authors:** Frosch Y. X. Quek, Nadyanna M. Majeed, Meenakshi Kothari, Verity Y. Q. Lua, Hee Seng Ong, Andree Hartanto

**Affiliations:** School of Social Sciences, Singapore Management University, 90 Stamford Road, Singapore 178903, Singapore; froschquek@smu.edu.sg (F.Y.X.Q.); nadyannam.2017@smu.edu.sg (N.M.M.); meenakshik.2018@smu.edu.sg (M.K.); verity.lua.2018@smu.edu.sg (V.Y.Q.L.); heesengong@smu.edu.sg (H.S.O.)

**Keywords:** brief mindfulness intervention, working memory capacity, focused breathing, operation span task, symmetry span task

## Abstract

Mindfulness training has been extensively researched and found to elicit positive effects on cognitive performance, including working memory capacity. Benefits to cognitive function have been extended to brief mindfulness training as well. However, not much is known about its effect on working memory capacity. The current study examined the effects of a single 15-min session of mindful attention to breathing compared to a 15-min session of mind-wandering exercise in a within-subjects experimental design (Study 1; *N* = 82) and a between-subjects experimental design (Study 2; *N* = 424). Contrary to our hypotheses, in both experiments, we did not find any evidence that participants in the mindfulness condition outperform the control condition on an operation span task (Study 1) and a symmetry span task (Study 2). These results suggest that a single session of mindful practice may not be sufficient to enhance working memory capacity.

## 1. Introduction

Mindfulness, which is broadly conceptualized as a concentrated and present-centered awareness (of thoughts), comprises mainly two components: self-regulated attention and openness to accepting present experiences [1,2]. As such, the cultivation of mindfulness is often through the involvement of high awareness and higher-order cognitive processes such as attention and working memory [3]. Accordingly, the practice of mindfulness which engages attention, working memory, and monitoring of mind-wandering would be expected to simultaneously train and strengthen these same cognitive processes [4]. At the same time, the state of mindfulness, being non-elaborative and non-judgmental, observes thoughts and feelings as events without over-identifying with them, and thus propagates a dispassionate state of self which is noted to enhance executive functioning [5,6,7]. Indeed, in numerous studies, mindfulness training has been shown to elicit positive effects on cognitive functions [8,9,10,11,12]. More specifically, the literature suggests a strong connection between mindfulness exercises and a key executive function, working memory capacity.

A component of executive function, working memory is a system of storing and rehearsing information, coupled with the attentional regulation of the active portion of memory [13]. This system contributes to higher-order processing, decision-making, and is highly associated with fluid and crystallized intelligence, traditionally known to represent the ability to reason and the competency of knowledge, respectively [14]. Working memory can be quantified by the working memory capacity, which indicates how well an individual is able to control their attention to maintain more information as active [13,15]. Given that mindfulness operates through sustained attention towards a specific set of stimuli, it is expected that training in meditation entails a boost in working memory capacity [4]. For instance, a study by Jha and colleagues [16] supports this notion by demonstrating that military men who had spent more time practicing mindfulness meditation during an eight-week training program performed significantly better on working memory tasks, compared to their peers who had spent less time or had not been trained in meditation at all. In another study by van Vugt and Jha [17], participants who had participated in a month of intensive mindfulness training, which required 10–12 h of daily practice, displayed better performance on working memory tasks than their counterparts in a control group without training. Overall, studies have extensively shown the positive impacts on working memory capacity in subjects who have undergone intensive mindfulness training compared to controls who have not [18,19].

Besides evidence from intensive mindfulness training, studies examining the benefits of brief, single-session mindfulness training on cognitive performance have been optimistic [20]. Supporting this notion was a study that found that undergraduate students who were engaged in a 15-min mindful breathing exercise performed better on a reading comprehension task than their counterparts who were assigned to a mind-wandering exercise [21]. Furthermore, Mrazek and colleagues [22] observed in a study using an 8-min mindful breathing exercise, that compared to students in a relaxation and reading control group, students in the treatment condition displayed reduced mind-wandering during a sustained attention task. Despite the brevity, these studies have demonstrated the efficacy of quick and easily accessible mindfulness training, suggesting that such a short-term mindfulness practice could be beneficial for training working memory as well. Research pertaining to brief mindfulness exercises and their effect on working memory capacity is, however, scarce. 

To test the effect of brief mindfulness on working memory, we conducted two pre-registered experimental studies employing two recordings: a mindful breathing exercise or a mind-wandering exercise spanning 15 min each (developed by Hafenbrack et al., adapted from Clinton et al. and Arch and Craske [21,23,24]). Experimental manipulation was performed by randomly assigning participants to listen to either 15 min of mindful breathing instructions or 15 min of mind-wandering instructions (i.e., control). With evidence from prior work, we hypothesized that subjects who underwent the 15-min mindful breathing exercise would perform better than subjects who underwent the 15-min mind-wandering control. 

## 2. Study 1

Study 1 employed a within-subjects approach to examine the effect of a brief mindfulness intervention on working memory capacity. By accounting for interindividual variability, the within-subject experimental approach has strengths in increasing power and minimizing error rates caused by individual differences [25,26]. To reduce carry-over effects—a potential confound—we utilized counterbalancing and required participants to undergo both the intervention and control conditions at two separate time points, one week apart. Testing the effects of the conditions one week apart allowed for any training or placebo effects to lose salience before carrying out another test, thus reducing carry-over effects.

### 2.1. Materials and Methods

#### 2.1.1. Participants

A total of 89 undergraduates from a local university in Singapore participated for either extra course credit or cash compensation. As per our pre-registered data collection plan, seven participants were excluded as they failed the attention check embedded in the study or scored less than 65% on the distractor trials in the working memory task. The final sample thus, consisted of 82 participants (71.95% female). Descriptives were reported only for participants who were included in analyses and are presented in Table 1. All participants gave informed consent to participate in the study prior to the onset of the experiment. Data collection was approved by the local IRB [IRB-20-123-A078(1020)]. Details of the pre-registration are available on AsPredicted at https://aspredicted.org/de2qp.pdf. Due to restrictions imposed by the COVID-19 pandemic, the planned recruitment of 150 participants was not achieved and the pre-registered sample size was not met.

#### 2.1.2. Procedure

Study 1 involved a within-subjects design where participants were randomly assigned using a random number generator to one of the two conditions in their first session and were thereafter assigned to the other condition in a second session one week later. To minimize order effects, the order of the conditions was counterbalanced across all participants such that half the participants completed the mindfulness condition first, and the other half completed the active control condition (mind-wandering) first. The study was conducted online via Qualtrics and participants were notified via email, 10–15 min prior to their registered timing, with instructions and the link to the online study.

Following the informed consent, participants in the mindfulness condition heard a 15-min audio track on a mindfulness breathing exercise. The track included directions such as “Focus your awareness on the sensations of slight stretching as the abdomen rises with each in-breath and of gentle deflation as it falls with each out-breath”. Participants were asked to direct all their attention to the present, feel their breathing and be mindful of their bodily sensations. Conversely, participants in the control condition were instructed to let their minds wander in the 15-min audio track. Directions such as “Now simply think about whatever comes to mind, let your mind wander freely [...] just let your mind roam as it normally would”, with no mindfulness advice given to the participants so that they would have an unrestricted flow of thoughts. To test whether participants paid attention to the audio track, participants were asked to respond to a listening check after listening to each track. Participants were asked to describe the content of their respective audio tracks in one or two sentences.

As an exploratory outcome, stress was measured after exposure to the intervention via three items (stress, worry, and calm) on a five-point scale (1 = Not at all, 5 = Extremely). The item “calm” was reverse-coded. The mean of the three items was calculated to arrive at a stress level for each participant in each condition (α = 0.77). In view of strong evidence for mindfulness practices reducing stress in healthy individuals [28], stress was included as an outcome of interest and acts only as an exploratory item, hence it was not reported during pre-registration of this study.

**Figure 1:**
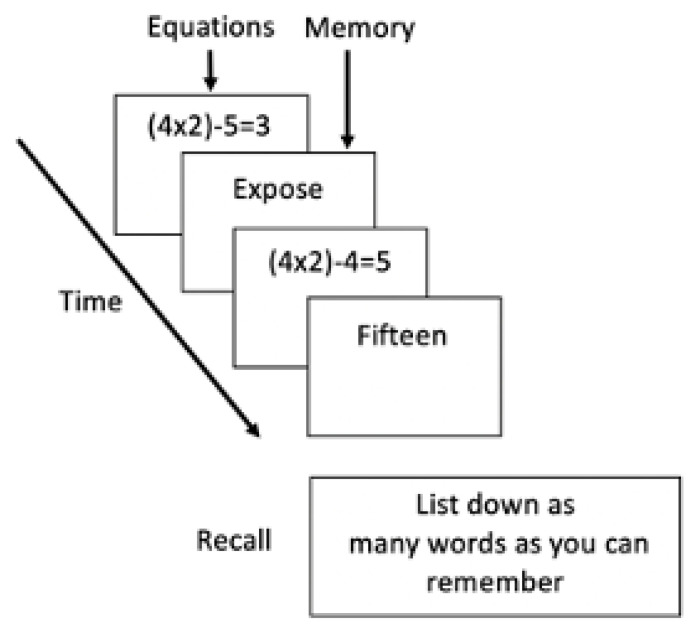
Operation Span Task.

The main outcome of interest, working memory capacity, was assessed via the operation span task [29,30]. As shown in Figure 1, participants evaluated mathematical equations (e.g., (3 × 3) − 3 = 6) while memorizing words up to three syllables long for later recall. Each word was presented right after each equation. Each set consisted of four to six equation-word pairs, thus resulting in three possible set sizes. The syllables in the mindfulness condition and active control condition were matched for lexical and semantic characteristics that may affect working memory recall, such as the number of letters, word frequency, orthographic and phonological neighborhood, familiarity, emotional valence, lexical decision latency, concreteness rating, and age of acquisition [31], all *ps* > 0.05. At the end of each set of equation-word pairs, participants were asked to recall as many of the words from the set as possible. Working memory capacity was then evaluated by the partial credit unit score (α_session1_ = 0.84, α_session2_ = 0.84) [32], which was calculated by dividing the number of correctly recalled words by the total number of words presented for each set. Prior to the actual task, participants completed two trial blocks of three equation-word pairs each to familiarize themselves with the task.

To reduce bias from poor data quality, participants were then faced with an attention check question that involved a distractor preamble about bilingual research but instructed participants to show that they were paying attention by choosing the option “Other” to continue. Additionally, they were asked if they provided honest responses throughout the course of the study. Lastly, demographic variables including sex, socio-economic status, age, and education level were collected at the end of the second session.

### 2.2. Results

All analyses were conducted in R version 3.6.3 [33]. Bayesian hypothesis testing was conducted using BayesFactor version 0.9.12–4.2 [34] in R, with Jeffreys-Zellener-Siow (JZS) priors. Standardized group mean difference effect sizes were calculated in terms of Becker’s *d* for within-subjects designs [35]. A summary of the results can be found in Table 2.

#### 2.2.1. Working Memory Capacity

**Figure 2:**
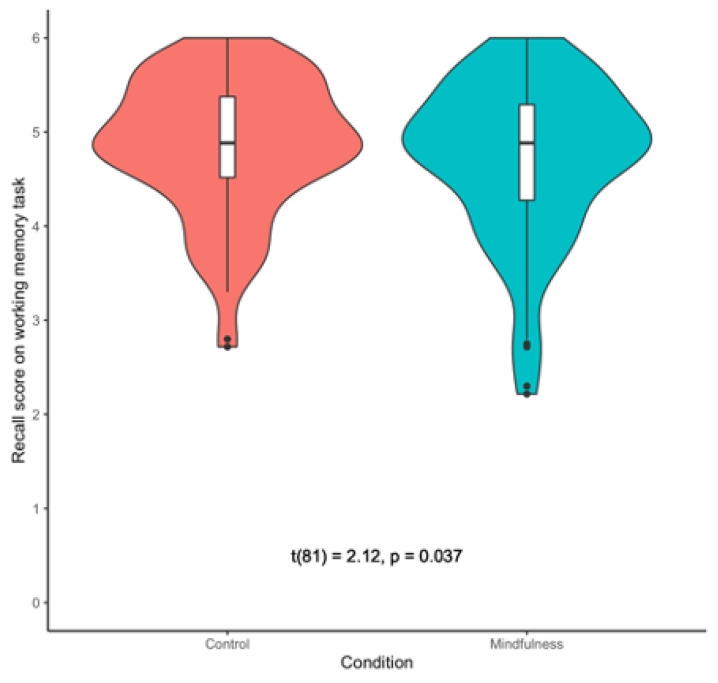
Violin Plots Depicting Working Memory Capacity in Study 1.

Following our pre-registered analytic plan, we tested if there was a difference in working memory capacity between the mindfulness and control conditions using both frequentist and Bayesian two-tailed paired-samples *t*-tests. The frequentist analysis revealed a significant difference in working memory capacity between the two conditions, *t*(81) = 2.12, *p* = 0.037, *d* = −0.19. However, the direction of the effect was contrary to our prediction—we found that participants performed better in the control condition (M = 4.85, SD = 0.72) than in the mindfulness condition (M = 4.71, SD = 0.84). These results are depicted in Figure 2. Nevertheless, the Bayesian t-test revealed only anecdotal support for the alternative hypothesis, BF_10_ = 1.01.

#### 2.2.2. Exploratory Outcomes

For exploratory purposes, due to the unbalanced distribution of sex in the current sample, we conducted a repeated-measures ANOVA with sex entered as a covariate to examine if there still was a difference in working memory capacity between the mindfulness and control conditions after controlling for sex. We found no main effect of condition, *F*(1, 80) = 2.36, *p* = 0.128, η_p_^2^ = 0.029. Additionally, we found no interaction between condition and sex, *F*(1, 80) = 0.25, *p* = 0.621, η_p_^2^ = 0.003.

Additionally, we examined if there was a significant difference in stress between the two conditions. A two-tailed paired-samples t-test revealed no significant difference, *t*(81) = 0.57, *p* = 0.570, *d* = −0.06, suggesting that mindfulness and the control condition (mind-wandering) did not have different effects on stress in the current sample.

## 3. Study 2

In order to address concerns about carry-over effects as well as the relatively small and age-restricted sample in Study 1, we conducted a follow-up study using a between-subjects design with a larger community sample in Study 2. Participants were randomly assigned to one of the two conditions, with the same manipulation as in Study 1. 

### 3.1. Materials and Methods

#### 3.1.1. Participants

A total of 493 participants were recruited via Amazon’s Mechanical Turk (MTurk), an online crowdsourcing platform, with cash compensation. As per our pre-registered data collection plan, 11 participants’ responses were discarded as they failed the attention checks embedded in the study, and an additional 59 participants’ responses were removed as they scored less than 65% on the distractor trials in the working memory task. The final sample hence consisted of 424 participants (56.25% female). Descriptives are reported for participants who are included in analyses and are available in Table 3. All participants gave informed consent to participate in the study prior to the onset of the experiment. Data collection was approved by the local IRB [IRB-20-123-A078-M1(1120)]. Details of the pre-registration are available on AsPredicted at https://aspredicted.org/7jx5d.pdf.

#### 3.1.2. Procedure

Study 2 involved a between-subjects design where participants were randomly assigned via Qualtrics to either the mindfulness condition (15-min mindfulness breathing exercise) or the active control condition (15-min mind-wandering exercise). Similar to Study 1, the study was conducted online via Qualtrics. 

In light of the monetary compensation and anonymity of MTurk studies, the study was only open to MTurk workers from the United States who had completed at least 500 Human Intelligence Tasks (HITs) and had at least a 99% HIT approval rating to ensure that high-quality data was collected. In addition, to prevent bots from providing fake data, a “Botcha” screener was implemented. Participants first had to answer two questions to determine that they were real human beings instead of programmed bots. Participants who failed any of the two questions were not directed to the main study and therefore their surveys were prematurely terminated with no remuneration. Following this was an audio check to ensure the smooth delivery of the manipulation without technical issues, and participants then gave informed consent before proceeding to the study properly. 

Before proceeding to the main study, participants were asked an attention check question similar to that in Study 1, where they had to show their attention by clicking on “Other” as the answer to a question after reading a preamble. Subsequently, baseline stress was measured via three items (stress, worry, and calm) on a five-point scale (1 = Not at all, 5 = Extremely). The item “calm” was reverse-coded. The mean of the three items was computed for each participant to arrive at a baseline stress score (*α* = 0.81).

Similar to Study 1, participants assigned to the mindfulness condition listened to an audio track instructing them to direct all their attention to their breathing and bodily sensations. Conversely, participants in the active control condition were instructed to allow their minds to wander freely with no mindfulness advice. To ensure the involvement of the participants in listening to the audio track there was a listening check for each condition. Participants were asked to describe the content in their respective audio tracks in one or two sentences. 

Afterward, as a manipulation check, absorption was measured via one item, “After listening to the recording, how much do you feel absorbed in the present moment?”. Participants rated their absorption on a seven-point scale (1 = Not at all absorbed, 7 = Extremely absorbed). Per Study 1, stress was a secondary outcome of interest. Stress was measured post-manipulation via the same three items (stress, worry, calm) on a five-point scale as at baseline. The mean of the three items was computed for each participant to arrive at a post-manipulation stress score (*α* = 0.80). The post-manipulation score was then subtracted from the baseline score for each participant to ascertain the decrease in stress for each participant. Both the absorption and stress outcomes were not included in the pre-registration analytic plan due to their secondary roles as either a manipulation check or an exploratory outcome.

**Figure 3:**
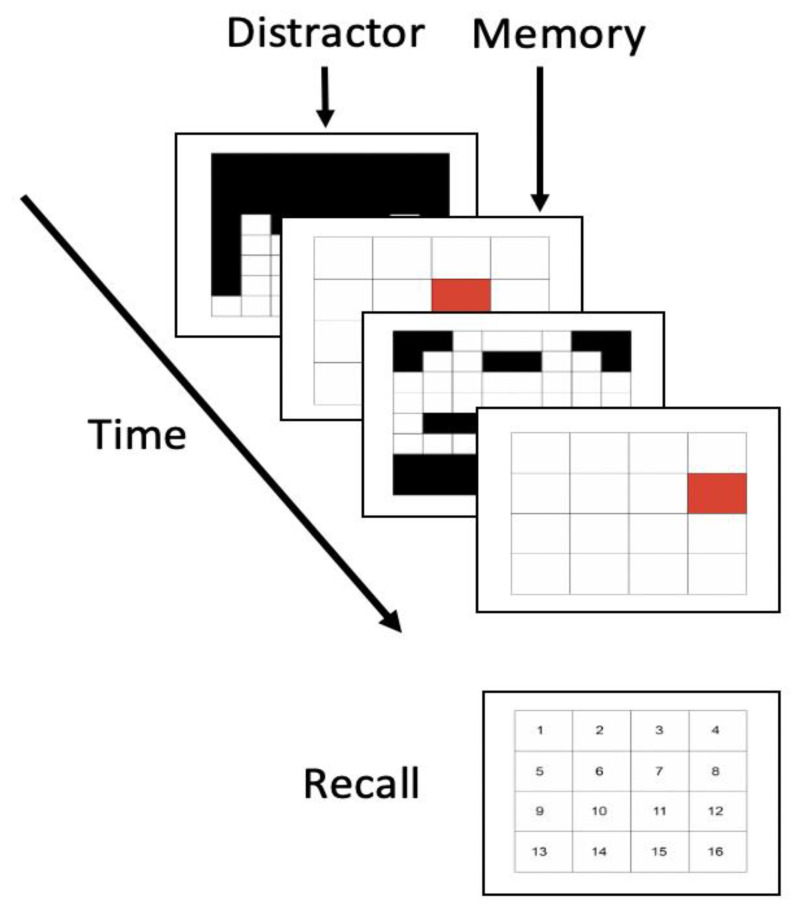
Symmetry Span Task.

The outcome of interest, working memory capacity, was assessed by the symmetry span task [36,37] in Study 2. As shown in Figure 3, participants were shown a series of grid locations one-by-one forming the 4 × 4 location grid in the center of the screen. Before each location grid was shown, the participant was shown a distractor in the form of an 8 × 8 symmetry grid. The pattern was either symmetrical or asymmetrical along its vertical axis, and the participant was to make a judgment (i.e., “Is this image symmetrical along the vertical axis?”) before the next 4 × 4 location grid was shown. At the end of each symmetry-location block, participants were asked to recall as many grid locations as possible in the order of appearance. Similar to Study 1, working memory capacity was then evaluated by the partial credit unit score (*α* = 0.85) [32]. Prior to the actual task, participants completed two trial blocks of three symmetry-location pairs each to familiarize themselves with the task. 

As in Study 1, participants were asked an honesty question to ascertain data quality. Lastly, participants were asked about their demographic characteristics including their sex, socio-economic status, age, and education level. A unique compensation code was generated for each participant which was used in the MTurk platform to collect their study compensation. 

### 3.2. Results

As in Study 1, all analyses were conducted in R version 3.6.3 [33]. Bayesian hypothesis testing was conducted using BayesFactor version 0.9.12–4.2 [34] in R, with Jeffreys-Zellener-Siow (JZS) priors. Standardized group mean difference effect sizes were calculated in terms of Cohen’s *d* for between-subjects designs [38]. A summary of the results can be found in Table 4.

#### 3.2.1. Working Memory Capacity

**Figure 4:**
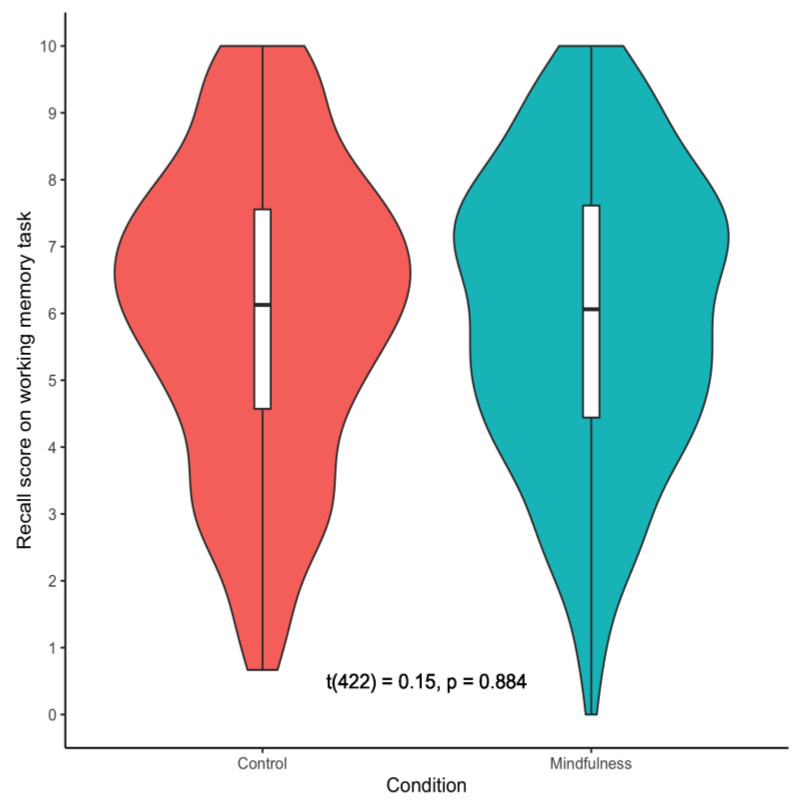
Violin Plots Depicting Working Memory Capacity in Study 2.

Following our pre-registered analytic plan, we tested if there was a difference in working memory capacity between the mindfulness and control conditions using both frequentist and Bayesian two-tailed independent-samples *t*-tests. In contrast to our findings in Study 1, the frequentist analysis revealed no significant difference in working memory capacity between the mindfulness and control condition in Study 2, *t*(422) = 0.15, *p* = 0.884, *d* = −0.01. These results are depicted in Figure 4. In line with the results of the frequentist analysis, the Bayesian t-test revealed substantial support for the null hypothesis (i.e., there is no significant difference in working memory capacity between the two conditions), BF_10_ = 0.11.

#### 3.2.2. Exploratory Outcomes

The absorption measure was used as a manipulation check. A two-tailed independent-samples t-test revealed that the difference between the two conditions was significant, *t*(422) = −2.02, *p* = 0.045, *d* = 0.20, such that participants in the mindfulness condition (M = 4.07, SD = 1.19) were more absorbed than those in the control condition (M = 3.83, SD = 1.25), suggesting that the manipulation was successful. 

A two-tailed independent-samples t-test was conducted to examine if participants in the two conditions differed in how much their stress decreased. There was a significant difference in stress reduction between the two conditions, *t*(422) = 2.40, *p* = 0.017, *d* = 0.23. Participants in the mindfulness condition (M = 0.37, SD = 0.71) experienced greater reductions in stress than participants in the control condition (M = 0.21, SD = 0.68). 

## 4. Discussion

The present studies aimed to examine if a brief mindful breathing exercise would improve the performance of individuals on operation and symmetry span tasks, which are measures of working memory capacity. The practice of brief mindfulness in our studies entailed a persistent effort to maintain focus on a single experience, particularly the sensation of breathing, for 15 min, and guidance to perform this intervention was through a pre-recorded audio track. Findings from both the within-subjects and between-subjects experiments showed no support for the studies’ hypothesis. Additionally, using a Bayesian t-test, the null hypothesis was shown to be substantially supported in Study 2. Despite earlier positive findings that mindfulness training enhances working memory [9,16,17,39,40], the null findings of the present studies suggest that 15 min of brief mindful breathing exercise does not elicit improvements in working memory capacity. 

This is in line with past evidence showing that brief mindfulness training may not always improve cognitive performance, or more specifically working memory [41,42,43]. For instance, Johnson and colleagues [42] found that a single 25-min session of mindful attention to breathing did not correspond to significant differences in cognitive task performance measured right before and after the task compared to a single 25-min session of relaxation and deep breathing. Incidentally, findings from both the present studies and Johnson et al. suggest that emotions or mood are more likely to be influenced by a short session of mindful breathing, as represented by measures of stress or anxiety. Although the previous work on mindfulness largely found support for the positive influence of mindfulness training on working memory, it is plausible that a brief session of mindful breathing is simply not enough to train the sustained attention and concentration required in a cognitively demanding task such as the complex span tasks used in this experiment [42]. Drawing from the findings of Basso and colleagues [41], whereby four weeks of mindfulness training (13 min daily) did not demonstrate improvements in working memory compared to eight weeks of training (13 min daily) which did, a likely conclusion is that single sessions of mindful practice are only effective when practiced regularly, over an extended period of time (i.e., at least more than four weeks). 

The current studies are not without their limitations. First, the studies only employed a single paradigm of working memory, the complex span task, and its null finding may not be generalizable to other working memory tasks (e.g., N-back task, digit span task, reading span task). Indeed, in Zeidan et al.’s [43] study, participants who underwent four separate sessions of 20-min meditation training were observed to have improved performance on the N-back task but not the backward digit span task which are both measurements of working memory capacity. As such, the present study’s conclusion on the ineffectiveness of brief mindfulness breathing exercise on working memory may be task-specific. It is therefore worthwhile for future studies to consider replicating the current study with different working memory paradigms such as the N-back [44,45], reading span, and other span tasks [32,36]. Similarly worthwhile would be for future studies to explore the effects of mindfulness practices on other domains of cognitive functions such as task-switching, inhibitory control, and episodic memory [46,47,48], which were not examined here. Second, the manipulation check adapted from Clinton et al. [21] is a single-item self-report measure and may have contributed to an overestimation of the effect of manipulation. Future studies may want to consider engaging other methods of manipulation checking that could more objectively determine the state of mindfulness in participants. Third, despite effective manipulation as indicated by the successful manipulation check in Study 2, the intervention could be transient and may not have lasted throughout the working memory task. Future studies could incorporate manipulation boosters and more manipulation checks to ensure that the effect of mindfulness remains salient throughout the course of the study. Lastly, while measures such as bot detection, attention checks, and honesty checks were taken, it is plausible that the online setting could lead to questions about the stringency at which participants engaged in the experiment. Additionally, the reliability of online experiments has been scrutinized, especially for more complex manipulations [49]. It would, therefore, be beneficial to consider providing more guidance in performing the interventions to ensure the effectiveness of the manipulation in future studies. Nonetheless, an effective manipulation was found using manipulation checks in the present studies and the results presented are still relevant.

## 5. Conclusions

In summary, the present study did not corroborate previous work on intensive mindfulness training, in that no evidence was provided to show the benefits of practicing brief mindful breathing on working memory. Although intensive mindfulness training has been largely found to enhance working memory [9,16,17,39,40,50], it may be argued that single-session mindfulness practices are too brief to elicit a significant positive impact on higher-order cognitive processes such as working memory.

## Figures and Tables

**Table 1:** Participant Characteristics in Study 1.

Characteristic	*N*	M (SD) or %	Range
Sex (% female)	82	71.95%	
Race (% Chinese)	82	80.49%	
Age (years)	81	21.02 (1.82)	18–27
Education ^1^	82	3.65 (0.78)	3–5
Household income ^2^	80	4.55 (2.30)	1–9
Personal income ^3^	82	1.79 (1.17)	1–9
Subjective socioeconomic status ^4^	82	6.27 (1.37)	3–10

Note. ^1^ Education was measured on a 6-point scale (1 = Below Secondary, 6 = Masters/PhD/Other Post-graduate degree). ^2^ Household income was measured using a nine-point scale in SGD 2500 intervals (1 = less than SGD 2500, 8 = more than SGD 20,000). ^3^ Personal income was measured on a nine-point scale in SGD 500 intervals (1 = SGD 0, 9 = more than SGD 3500). ^4^ Subjective socioeconomic status was measured using a 10-point ladder scale adapted from Adler et al. [27], with the bottom rung representing the lowest status and the top rung representing the highest status.

**Table 2:** Summary of Outcomes in Study 1.

Outcome	*N*	Mindfulness M (SD)	Control M (SD)	*t*	*df*	*p*	*d*
Working memory capacity	82	4.71 (0.84)	4.85 (0.72)	2.12	81	0.037	−0.19
Stress	82	2.58 (0.86)	2.63 (0.79)	0.57	81	0.570	−0.06

**Table 3:** Participant Characteristics in Study 2.

	Mindfulness (*N* = 208)		Control (*N* = 216)	
Characteristic	M (SD)	Range	M (SD)	Range
Sex (% female)	56.25%		57.41%	
Race (% White)	81.25%		78.70%	
Age (years)	42.90 (13.45)	19–73	44.58 (13.13)	19–77
Education ^1^	8.43 (2.08)	3–12	8.45 (2.02)	3–12
Household income ^2^	2.82 (1.72)	1–9	2.86 (1.92)	1–9
Personal income ^3^	6.06 (2.69)	1–9	5.68 (2.74)	1–9
Subjective socioeconomic status ^4^	5.02 (1.72)	1–10	5.10 (1.68)	1–9

Note.^1^ Education was measured on a 12-point scale (1 = No school or some grade school, 12 = PhD, ED.D, DDS, LLD, JD, or other professional degree). ^2^ Household income was measured using a nine-point scale in USD 2500 intervals (1 = less than USD 2500, 9 = USD 20,000). ^3^ Personal income was measured on a nine-point scale in USD 500 intervals (1 = USD 0, 9 = more than USD 3500). ^4^ Subjective socioeconomic status was measured using a 10-point ladder scale adapted from Adler et al. [27], with the bottom rung representing the lowest status and the top rung representing the highest status.

**Table 4:** Summary of Outcomes in Study 2.

Outcome	Mindfulness (*N* = 208) M (SD)	Control (*N* = 216) M (SD)	*t*	*df*	*p*	*d*
Working memory capacity	5.98 (2.16)	6.01 (2.22)	0.15	422	0.884	−0.01
Absorption	4.07 (1.19)	3.83 (1.25)	−2.02	422	0.045	0.20
Decrease in stress	0.37 (0.71)	0.21 (0.68)	2.40	422	0.017	0.23

## Data Availability

Publicly available datasets were analyzed in this study. This data can be found here: https://researchbox.org/170.
